# History of Assisted Reproductive Technology and Chlamydia Trachomatis Infection in Pregnancy

**DOI:** 10.14740/jocmr2444w

**Published:** 2016-01-26

**Authors:** Masahiko Kato, Shunji Suzuki

**Affiliations:** a Department of Obstetrics and Gynecology, Japanese Red Cross Katsushika Maternity Hospital, Tokyo, Japan

**Keywords:** Chlamydia trachomatis, Assisted reproductive technology, Pregnancy, Japan

## Abstract

**Background and Methods:**

To identify whether or not a history of assisted reproductive technology (ART) is associated with the low incidence of Chlamydia trachomatis (CT) infection in pregnant women, we reviewed the obstetric records of the Japanese women aged 35 - 42 years.

**Results:**

The prevalence of CT in the pregnancies without ART was 1.1% (28/2,632) using nucleic acid amplification tests, while it was zero in the pregnancies conceived by ART (0/364, P = 0.049 by the X^2^ test).

**Conclusions:**

A history of ART seemed to be a negative risk factor for CT infection in pregnant women.

## Introduction

Chlamydia trachomatis (CT) infection in pregnancy can cause maternal disease, adverse pregnancy outcomes, and neonatal disease [1]. In Japan, almost pregnant women are routinely tested for CT using nucleic acid amplification tests according to the guidelines for obstetrical practice in Japan for the prevention of neonatal CT infection [2]. The CT prevalence rate in the Japanese pregnant women was observed to be 2.4% with even higher rates among pregnant women teenaged at the time of delivery (about 16.0%), while the CT prevalence in the women aged ≥ 35 years was very low (0.8-1.0%) [3].

On the other hand, recently a dramatic increase in pregnancies conceived by assisted reproductive technology (ART) has been observed in Japan. According to the Annual report of Perinatology Committee in the Japan Society of Obstetrics and Gynecology (JSOG) in 2015 [4], the rate of pregnancies conceived by ART in the registration facilities of JSOG has been reported to be 7.3% in 2013 (13,573/186,234; *in vitro* fertilization and embryo transfer: 10.655, intracytoplasmic sperm injection: 2,918). This trend may have been contributed to the low prevalence of CT in the elderly pregnant women, because they have become pregnant without sexual intercourse.

Therefore, this study was examined to identify whether or not a history of ART is associated with the low incidence of CT infection in pregnant women.

## Methods

We reviewed the obstetric records of the Japanese women aged 35 - 42 years who had deliveries at Japanese Red Cross Katsushika Maternity Hospital from 2010 through 2014.

## Results

The prevalence of CT in the pregnancies without ART was 1.1% (28/2,632) using nucleic acid amplification tests, while it was zero in the pregnancies conceived by ART (0/364, P = 0.049 by the X^2^ test).

## Discussion

Based on the results, pregnancy conceived by ART may reduce the breaks of infection of CT at pre-pregnancy. Otherwise, there may be some factors to reduce the prevalence of CT infection in the backgrounds of women who required infertility therapy with ART.

Some other possible reasons or limitations associated with the result can be proposed. One may be the small sample size of the current observation, because the prevalence of CT in this age group is very low. It is estimated that a definitive trial powered to detect a same difference in the prevalence would require approximately 1,560 patients equally divided in the control and ART groups (two-tailed α = 0.05, β = 0.2). The other is that the women conceived by ART may have received treatments for CT during their fertility therapy. Because the presence of fallopian tube obstruction is one of the main indications for ART [4], and CT infection is one of the main causes of the tubal obstruction [1, 5]. The check of CT infection by nucleic acid and/or antibody detection tests is one of the basic examinations on infertility therapy in Japan [5]; however, unfortunately we might have missed interview concerning the possibility of a treatment history of CT.

### Conclusions

We understand that the current result does not lead to the recommendations of ART to reduce CT infection; however, a history of ART seemed to be a negative risk factor for CT infection in pregnant women.
